# Mobile Devices for Community-Based REDD+ Monitoring: A Case Study for Central Vietnam

**DOI:** 10.3390/s130100021

**Published:** 2012-12-20

**Authors:** Arun Kumar Pratihast, Martin Herold, Valerio Avitabile, Sytze de Bruin, Harm Bartholomeus, Carlos M. Souza, Lars Ribbe

**Affiliations:** 1 Centre for Geo-Information, Wageningen University, P.O. Box 47, 6700 AA, Wageningen, The Netherlands; E-Mails: martin.herold@wur.nl (M.H.); valerio.avitabile@wur.nl (V.A.); sytze.debruin@wur.nl (S.B.); harm.bartholomeus@wur.nl (H.B.); 2 Institute for Technology and Resources Management in the Tropics and Subtropics (ITT), Cologne University of Applied Sciences, Betzdorfer Str. 2, 50679 Köln, Germany; E-Mail: lars.ribbe@fh-koeln.de; 3 Instituto do Homem e Meio Ambiente da Amazônia—Imazon, Caixa Postal 5101, Belém, PA 66613-397, Brazil; E-Mail: csouza608@gmail.com

**Keywords:** mobile devices, REDD+, MRV, community based monitoring, forest carbon, forest change

## Abstract

Monitoring tropical deforestation and forest degradation is one of the central elements for the Reduced Emissions from Deforestation and Forest Degradation in developing countries (REDD+) scheme. Current arrangements for monitoring are based on remote sensing and field measurements. Since monitoring is the periodic process of assessing forest stands properties with respect to reference data, adopting the current REDD+ requirements for implementing monitoring at national levels is a challenging task. Recently, the advancement in Information and Communications Technologies (ICT) and mobile devices has enabled local communities to monitor their forest in a basic resource setting such as no or slow internet connection link, limited power supply, *etc.* Despite the potential, the use of mobile device system for community based monitoring (CBM) is still exceptional and faces implementation challenges. This paper presents an integrated data collection system based on mobile devices that streamlines the community-based forest monitoring data collection, transmission and visualization process. This paper also assesses the accuracy and reliability of CBM data and proposes a way to fit them into national REDD+ Monitoring, Reporting and Verification (MRV) scheme. The system performance is evaluated at Tra Bui commune, Quang Nam province, Central Vietnam, where forest carbon and change activities were tracked. The results show that the local community is able to provide data with accuracy comparable to expert measurements (index of agreement greater than 0.88), but against lower costs. Furthermore, the results confirm that communities are more effective to monitor small scale forest degradation due to subsistence fuel wood collection and selective logging, than high resolution remote sensing SPOT imagery.

## 1. Introduction

Tropical forests play an important role in the global carbon cycle. Human-related destruction activities such as deforestation and forest degradation lead to significant emissions of greenhouse gases (GHGs) in the atmosphere, resulting in accelerated global warming [1–3]. To mitigate this, Reduced Emissions from Deforestation and Forest Degradation in developing countries (REDD+) has been put forward for negotiation under the United Nations Framework Convention on Climate Change [4–6]. The recently agreed REDD+ mechanism includes reducing deforestation and forest degradation, forest enhancement, sustainable forest management and conservation. Besides reduction of carbon emissions, REDD+ also provide co-benefits in terms of biodiversity and livelihoods of the local community [4,7]. Currently, several REDD+ financial mechanism have been suggested for forest carbon credits including market-based mechanisms, fund-based systems, and their combinations [8,9]. All of these mechanisms are likely to distribute credits based on the amount of emissions reduction and to raise local participation to support the sustainability of the REDD+ program.

In order to implement the REDD+ mechanism, each nation needs to setup an effective, efficient and sustainable Measuring, Reporting and Verification (MRV) system at national, sub-national and local levels [10–12]. Thus, it is important to prepare a national MRV system from reliable, robust, transparent, creditable datasets that contribute to effective implementation of REDD+. Remote sensing and national forest inventory are principal data sources used to calculate forest area change, rates of deforestation and degradation, and are hence used to establish baseline reference emission levels [13]. However, national forest inventories are often outdated and inconsistent because they lack adequate financial support as well as technical and skilled human resources to acquire and update the data. Community based monitoring (CBM) is an alternative and effective way to reduce costs and to increase the reliability of forest monitoring data [14–16]. CBM can play a useful role for monitoring locally-driven change activities and small scale forest degradation due to, for example, subsistence fuel wood collection, charcoal extraction and grazing in the forest [10,17,18]. The impacts of these activities are rarely captured accurately in national databases or observed in remotely sensed imagery [19]. Data acquired by communities can include reporting on incidence of change events as well as ground measurements on carbon stock changes, which are essential for REDD+ reporting at national level [7,20]. Furthuremore, CBM can also create a strong local commitment to protect forests and biodiversity augment the chance to succeed in REDD+.

Mobile devices such as smart phones and personal digital assistants (PDA) have shown great potential to increase the local participation in data collection processes and hence, contribute to the effective implementation of CBM. Practical experiences from developing countries, such as Nepal, Tanzania, Cameroon, India, Mexico have demonstrated that local communities can play an essential role in acquiring forest inventory data [7,21]. Compared to traditional paper based methods, mobile devices offer the following immediate advantages [22–24]:

1. Mobile devices support mobility *i.e.*, supporting community participants to immediately record the measurement in a digital system;
2. The implementation of mobile based schemes is becoming cost-effective and sustainable because of the low cost of the devices;
3. Mobile devices have the potential to signal recent forest changes, including area of change and type of disturbance in near real time.

Despite these potentials, mobile based systems for CBM are still exceptional and they face implementation challenges when used by minimally-trained local communities in a REDD+ context. Additionally, fitness for use of these applications in terms of interaction and local suitability is often overlooked [25–27]. Currently available open source technologies such as CyberTracker [26], EpiCollect [28], ODK [29] and Xform [30] facilitate implementation, but they do not directly address fitness for use.

The main objective of this paper is to present an integrated mobile device based data collection system that allows streamlining the community-based forest monitoring data collection, transmission and visualization process, in order to improve the usability, accuracy and reliability of national MRV schemes. Such a system aims to bridge the information gap at national-level and also facilitates timing and consistent reporting of forest monitoring data. A prototype was built based on pervasive computer technologies and on the open source Android platform. The system performance was evaluated at the Tra Bui commune, Quang Nam province, central Vietnam by assessing the technical suitability of the proposed system. Finally, this paper assesses how CBM could contribute to an independent data stream for REDD+ implementation programs by comparing the locally gathered data to expert (local, regional and national) field measurements and a high resolution SPOT remote sensing image.

## 2. Materials and Methods

### 2.1. System Requirement

To implement a proper CBM, the local community needs a reliable way to record, store and deliver the collected information to the central database server [12,31]. Accordingly, the system needs to satisfy both hardware and software requirements. The hardware for data gathering should enable maintaining consistency with existing national MRV systems. Since the remote sensing and forest inventory data include images and geo-location points, the system should incorporate a positioning system and a camera. Popular smartphones with GPS and camera operating on Android satisfy these demands. The usability requirements of the system are specific to the local user. These requirements include:

- Multi-language support;
- Multi-user for simultaneous use;
- Applicability in remote location;
- Voice recording as desired functionality;
- Local data storage facilities.

### 2.2. System Design

**Figure 1.**
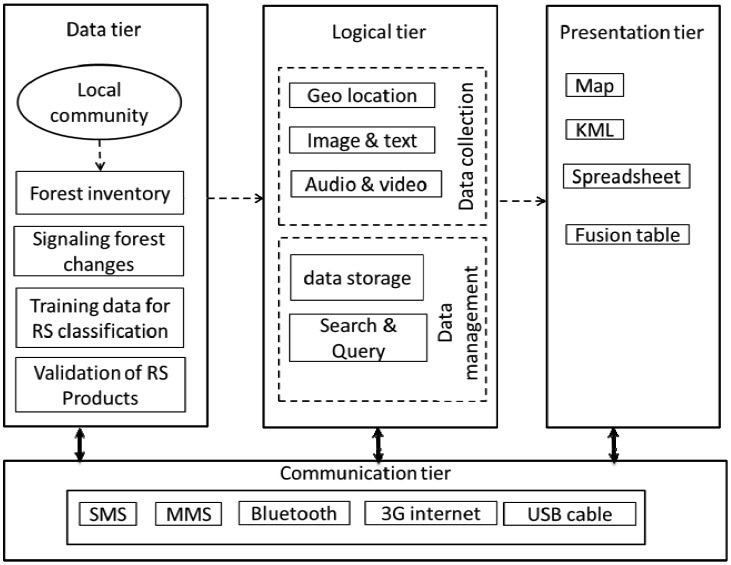
Service platform architecture for community based monitoring.

The proposed system provides a complete end-to-end platform which allows the local community to gather forest data and deploy forest measurements effectively. The general overview of the functional architecture of the system is outlined in Figure 1. The whole system is integrated into four-tier architecture: the data tier, logical tier, presentation tier and communication tier.

#### (a) Data Tier

**Figure 2.**
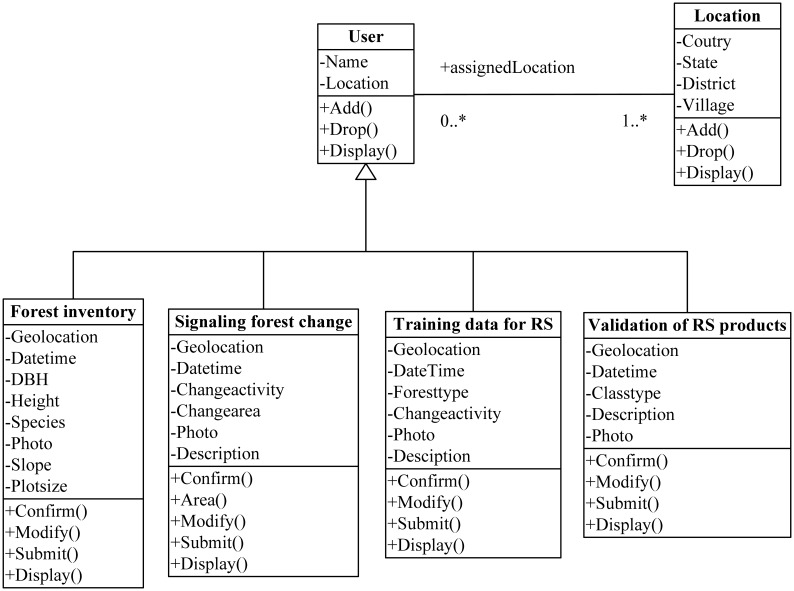
Class diagram of data acquisition form.

The data tier represents the data acquired at ground level. Figure 2 depicts a unified modeling language (UML) class diagram that illustrates the conceptual structure of the data acquisition system. Classes are portrayed as boxes with three sections: the top one shows the name of the class, the middle one lists the attributes of the class, and the third one lists the methods. The unfilled triangle shape (Δ) represents the aggregation among the classes. User class is designed as a root class which is associated with the following subclass: (1) forest inventory, (2) signaling forest change, (3) acquisition of training data for remote sensing classification and (4) validation data for remote sensing products. Many similarities in attributes and functions are designed to keep the data acquisition independent among these subclasses. A bi-directional association is showed by a solid line between the two classes, namely user and location. The user class is associated with a specific location, and the user class recognizes this association. The user takes on the role of “assignedLocation” in this association. The multiplicity value next to the user class of 0..* indicates that when an instance of a user exists, it can either have one instance of a user associated with it or no user associated with it (*i.e.*, maybe a user has not yet been assigned). In addition to that the location instance can be associated either with no user or with up to an infinite number of users.

#### (b) Logical Tier

The logical tier consists of overall system design units such as data collection and management units. The data collection component comprises optional input constraints such as text, image, audio/video, geo-location, flow depending on previous answers; icon based user-friendly graphics and local language support. Central data management is one of the major goals of the system. It facilitates data access and exploration for end users. Furthermore, Search tools allow users to search, query and modify particular data. The local data, upon meeting all the national requirements, can also be integrated into a third-party national or international database.

#### (c) Communication Tier

In order to avoid data loss, the proposed system allows for local storage of the collected data in an extensible markup Language (XML) format along with associated binary files (image, audio, and video;. These locally stored data can be transmitted to the server using thick and thin client-server architecture [25]. A thin client provides limited applications on the client and all the data storage and processing occurs on the server. In contrast, a thick client is embedded with the bulk of data storage and processing capacity. With thick clients, there is low level performance required from the server resulting in minimum server load and a faster response time. The communication tier allows users to synchronize with a data server at any time using any available data transmission means such as Short Message Service (SMS), Multimedia Messaging Service (MMS), Bluetooth, 3rd generation (3G) internet and Universal Serial Bus (USB) cable. Table 1 presents the data transmission means in terms of data type, data volume and cost.

#### (d) Presentation Tier

The presentation component intends to visualize the community collected data in a tabular and map form respectively. The tabular forms are spreadsheets, Comma Separated Values (CSV) format. Google fusion table whereas map forms allow visualisation in a static and in a dynamic mode through Hyper Text Markup Language (HTML) web browsers. Google Fusion Tables is an online data management application that facilitates easy collaboration, data visualization and web publishing [34].

### 2.3. System Implementation

**Figure 3.**
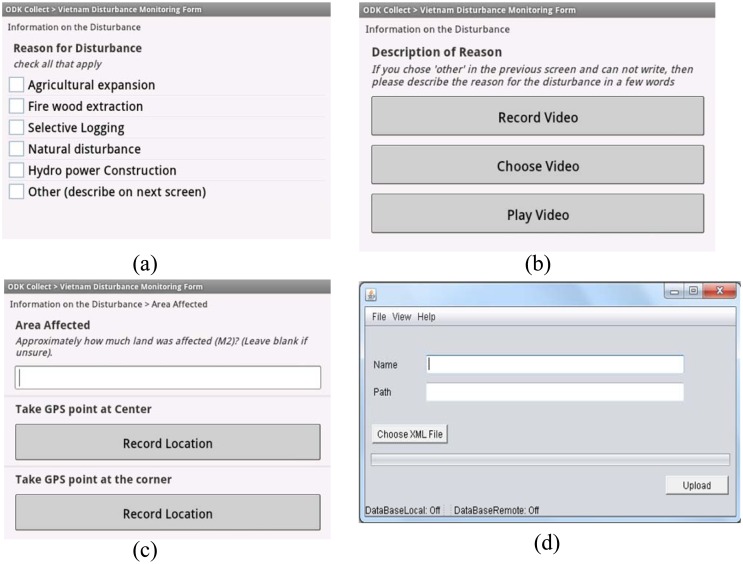
Some screenshots of deployed application interface to: (**a**) select the reason for forest disturbance; (**b**) record video as forest disturbance description; (**c**) record area for forest disturbance and (**d**) upload form to the database server.

The prototype was implemented in a hierarchical structure. Initially, the data tier described in Section 2.2 was implemented using the XML file format because it provides a flexible way to represent class attributes. Four types of forest monitoring forms were created in XML format, namely: forest inventory (for estimation of above ground forest biomass), signaling forest change (for reporting of forest disturbance), training data for remote sensing classification and for validation of remote sensing products. Different means of data input design interface were used to facilitate data entry, such as selection option, multimedia (audio/video), photographs and entering text, number, selection options, dropdown menus. The developed form is deployed at the client side (mobile platform) through ODK collect. Considering the fact that users may not always have access to an internet connection, thick and thin client-server architectures of data transmission systems were implemented. Local people can upload their data from the mobile device to a local data base through an USB connection or via SMS, MMS or Bluetooth. Consequently, the availability of an internet connection permits submission of monitored information to the remote server. Java 2 Enterprise Edition (J2EE) for java netbeans was used to provide interfaces to manage data collection forms and the collected data is stored in PostgreSQL. QuantumGIS linked with PostgreSQL, allows the data analysis operations on managed data, visualized in a map form. The web map server (WMS) plugin of QuantumGIS allows map processing through HTML web browsers. Similarly, the system also allows interoperability to deploy the data on different kinds of cloud computing environments such as Google app engine. Some screenshots of the deployed application are shown in Figure 3.

### 2.4. System Evaluation in the Case Study of Central Vietnam

#### 2.4.1. Field Setup

**Figure 4.**
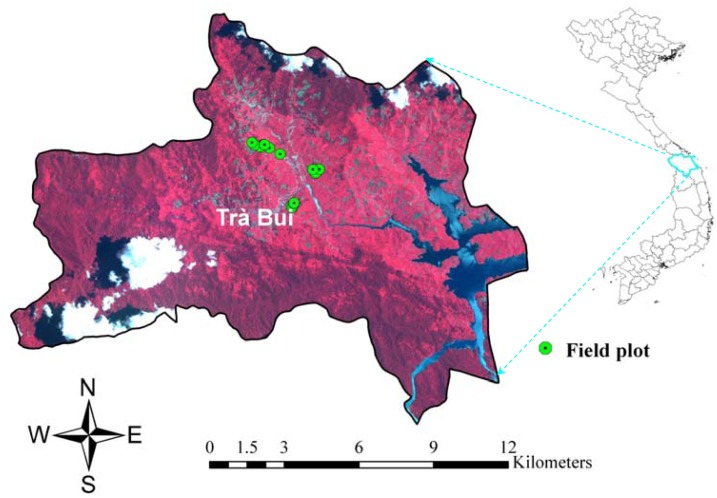
The study area overlaid with Spot remote sensing image and community measured field plots.

The capability of the developed system for monitoring the forest carbon and change activities was tested and evaluated at Tra Bui commune (15°17′54.65″N,108°08′08.01″E to 15°19′37.97″N, 108°08′13.42″E), Quang Nam province, central Vietnam (Figure 4). The forest of the Tra Bui commune area has been under threat since the construction of the Sông Tranh 2 hydroelectric dam, which caused the resettlement of the population of this community, mostly belonging to an ethnic minority. In order to regain land to cultivate crops the majority of the households have cleared parts of the forest. The following steps were carried out to deploy the prototype:

1. Initially, the questionnaires were designed based on the indicator required to assess the technical capacity of the community members. The questionnaires were used for the collection of data with the techniques of household surveys, interviewing commune leaders, and a participatory workshop based on Participatory Rural Appraisal (PRA) [35] smethods. More than 80 people were consulted during this process. In the meantime potential organizations were contacted for the collection of necessary documents for the research such as 3G coverage over the study area and the electricity supply time table.
2. User friendly training materials were produced in a local language for the developed technology and methods for acquisitions of data. Community training was conducted before implementation of the program. The training was meant to enhance the capacity of the community and to envision approaches and strategies for program implementation.
3. A purposive sampling design was used to evaluate the intellectual interaction of the system with the local community within a limited time [36]. Specific types of local knowledge such as accessibility and indicated forest change areas were used as sampling information. Circular biomass plot with 10 m radius were designed in a homogeneous forest area. Diameter at Breast Height (DBH) and tree species of all trees inside the plot were measured. Also, time required to enter the data was recorded. Similarly, forest disturbances were recorded around the disturbed area. A Samsung Galaxy tab 7.0 mobile device, a diameter tape and clinometer were used as measuring equipment.
4. During the implementation, paper based forms were also used in each location to enter the data. Data entry of each participant was compared to the paper based data entry. Furthermore, structured interviews were conducted with individual users to receive feedback regarding the data entry interface and overall performance of the system. In total 80 people were interviewed during this process.

#### 2.4.2. Comparisons

Two types of comparison data sets were acquired to evaluate the technical skills and measurement quality of the local community. Firstly, local experts (local forest rangers) and national experts (regional/national forest rangers) were trained with the system. Reference measurements were obtained by repeating the entire community measurements (17 biomass plots and 48 disturbance monitoring plots) by local experts. Due to cost constraints, only seven biomass plots and eight disturbance monitoring plots were repeated with national experts. Finally, above ground biomass was estimated from measurements made by local people and experts using biomass allometric equations [37]. The national forest inventory catalogue was used to covert local names of tree species to scientific names. Secondly, a time series of high resolution remote sensing images acquired between 2007 and 2011 (pan sharpened SPOT 5 images) were used for this research (Table 2), provided by Planet Action for the Land Use and Climate Change Interactions project (LUCCi) in the Vu Gia Thu Bon basin, central Vietnam [38]. Locally reported disturbance monitoring signals were visually interpreted based on the Pohl and Van Genderen [39] approach. Following this approach, images were systematically examined and forest disturbed pixels area manually digitized as polygons. The forest disturbed areas were estimated by calculating the polygon area.

### 2.5. Statistical Analyses

Comparisons between measurements of community and local expert values were carried out by simple linear regression where locally measured values were used as the dependent variable and the expert data were used as the independent variable. Linear correlation parameters (*r*) were calculated, which expresses the strength and the direction of a linear relationship between two variables. Additionally, the Index of Agreement (IA) was computed as described by Willmott [40] (Formula 1). IA is standardized measure of the degree of locally observed error and varies between 0 and 1. A value of 0 indicates no agreement at all and 1 indicates a perfect match [40]:

(1) $$\text{IA}=1\frac{\sum_{1=1}^{\text{n}}{\left({\text{y}}_{\text{i}}-{\text{x}}_{\text{i}}\right)}^{2}}{{\sum_{\text{i}=1}^{\text{n}}\left[\left|\left({\text{y}}_{\text{i}}-\bar{\text{x}}\right)\right|\left|\left({\text{x}}_{\text{i}}-\bar{\text{x}}\right)\right|\right]}^{2}}$$

where y_i_ is the i^th^ locally observed value, x_i_ the i^th^ expert measured value, x̄ is the mean of value and n is the number of observation.

## 3. Results and Discussion

### 3.1. Technical Capacity of the Local Community

The technical capacity of the local community turned out to be an important factor for deployment of the system. More than 60% of the local people use mobile phones as a daily means of communication. Among them, 12% use the mobile phone for calling, 30% for text messages and calling, 30% for calling, SMS and photography and 18% for calling, SMS, photography and internet.

### 3.2. System Performance

The results of system performance in combination with user interactions are reported in Table 3, which shows that the accuracy of data entries varies from user to user. Entering data via the text interface produced the highest error rates. Most of the errors were due to double entering of text and symbols on the electronic forms. The visual examination of all the captured multimedia was done manually. The result shows that 89% of captured multimedia by local community and 95% from local rangers have good quality. A common mistake during captured multimedia was that the targeted object was not highlighted correctly. Remarkably, the selection interface options presented no errors by all user types.

Cost is a critical variable for REDD+ implementation. This paper only considers the implementation cost of the system to monitor the forest. The device cost was not considered for the analysis. A simple cost analysis revealed that the community measurements costs were $1.20 United States Dollar per hectare (ha) whereas local expert costs were $3.2 per ha and the national expert cost $ 6.40 per ha (Table 3). Community measurements costs were significantly lower than expert-based measurements and they are expected to decrease as the size of sampled forest increases.

A major issue was data transmission because there was not enough coverage of the internet inside the forest. Only 15% of the plots had 3G internet coverage, so data storage was done locally on the mobile device. Data transmission from the mobile device to the local storage computer/laptop was done either using the 3G mobile connection or though the USB connection. Presence of wireless LAN connection allowed transmitting the data from the local level to the central database.

### 3.3. System Evaluation in Terms of REDD+ Implementation

**Figure 5.**
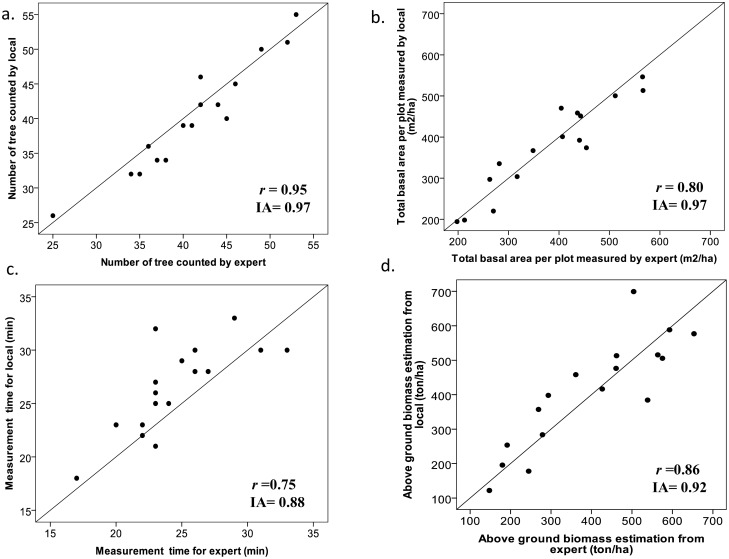
Simple linear correlation and error analysis statistics of the comparison between local (dependent variable y) and expert estimated (independent variable x) for: (**a**) Number of tree; (**b**) Total basal area per plot; (**c**) measuring time and (**d**) above ground biomass estimation. *r* is the linear correlation parameter, and IA is the index of agreement.

Figure 5 presents the results of the simple linear correlation and error analysis for comparison between local and expert (local forest rangers) measurements. All the indices of agreement are equal or higher than 0.88, indicating a good overall agreement between local and expert measurements. Figure 5(a) shows that the number of trees per plot was the parameter with highest agreement between local and expert measurements (IA = 0.97). Figure 5(b) indicates that there was good agreement between the total basal area per plot measured by locals and experts (points close to the 1:1 line) for all observations. The third observation (Figure 5(c)) is the difference in measurement time for local people and for expert. It can be observed that local people needed more time than the expert. Finally, the comparison of above ground biomass estimates (Figure 5(d)) showed that biomass estimation by experts was on average higher than that estimated by local people. One source of error was related to tree species identification, which was used to estimate wood density and tree biomass: local people always reported the tree species in local name but its conversion to the scientific name was often lacking in the national catalogue.

**Figure 6.**
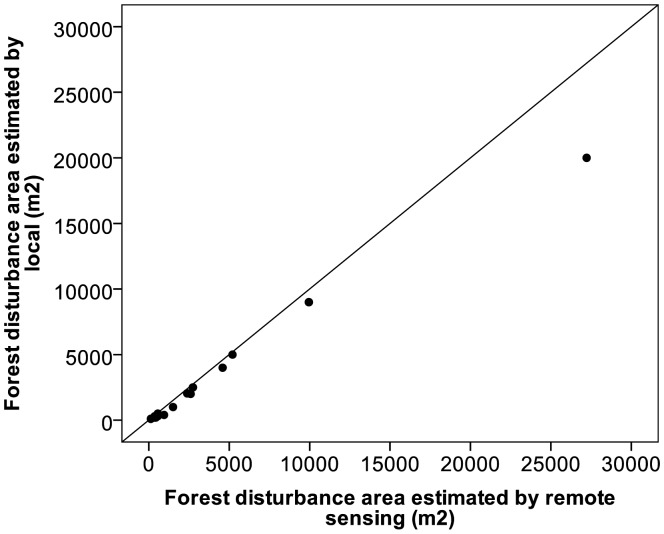
Relationship of forest disturbance area estimated by local and by SPOT remote sensing image.

However, REDD+ is not only about estimation of biomass but also about tracking forest disturbance, which provides an estimate of rates of deforestation and forest degradation. The number of forest disturbance events, their size and the timing of events are recorded by community members and were compared with remote sensing observations. Figure 6 shows the comparison of forest disturbance areas due to agriculture expansion captured by the local community and by remote sensing. The estimated areas show that there was high agreement for small and medium events but that local people underestimated a large area deforestation event compared to RS based estimates.

**Figure 7.**
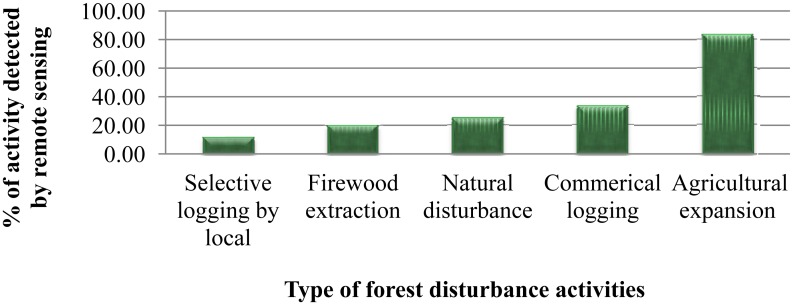
Percentage of locally reported forest disturbance types identified through SPOT image.

Similarly, Figure 7 shows the percentage of locally reported forest disturbance events identified through remote sensing. The result shows that only 18% of community-reported selective logging events can be visually identified using remote sensing, whereas around 88% of subsistence agricultural expansion was recognised in the satellite data, showing that local people are better able to identify the small scale forest disturbances.

Finally, remote sensing time series images were used to quantify the agreement with the disturbance date provided by the local community. Table 4 shows the delay in capturing forest disturbance signal by SPOT images. In general, the results show that a delay of 1 to 2 years can occur to capture events of disturbance and that between 14% and 36% of the events identified by the local communities were not detected in the SPOT images, highlighting the need of frequent ground monitoring. Nevertheless, the SPOT image of 2010 has captured 65% forest disturbance. The reason for this could be that the image was acquired immediately after the disturbance and most of the disturbances are due to agriculture expansion.

## 4. Discussion

### 4.1. Opportunity from a National REDD+ Perspective

From a national REDD+ monitoring and implementation perspective, it is important to involve local community groups and societies to carry out forest monitoring, in particular if there is any prospect of payment and credits for environmental services. This study evaluates the role of local communities in measuring above ground biomass and forest disturbance monitoring activities. The results show that the proposed system supports the idea of community based monitoring [31,41] enabling the capacity of a local community to monitor forest carbon and forest change activities effectively. Communities involvement allows the establishment of ‘ownership’ in forest management, strengthens their stake in the REDD+ reward system and greatly increase transparency in the sub-national/ national governance of REDD finances.

Generally, forest inventory is carried out on the national level to collect ground-based measurements (such as tree height, DBH and tree species) on plots selected through a sampling design, and uses these measurements to estimate forest carbon stocks using allometric relationships [37,42]. This process can be expensive and time consuming and few developing countries have comprehensive forest inventories that allow for national forest carbon stock estimates [43]. Experience gained from published studies shows strong agreement between local community and expert measurement in above ground biomass. Earlier studies conducted in Ghana, Tanzania, Nepal and Philippines [7,44,45] demonstrated that communities can collect some local forest inventory data adequately and at reduced cost than professional foresters. The results showed that local communities can measure and report the basic tree variables such as DBH, tree species, and tree count; and most importantly, they can repeat the measurements on a regular basis. The collected data has proven to be of a level of precision comparable to that produced by professional forest inventory staff.

SPOT remote sensing data of 2.5 meters ground resolution has difficulties to capture small scale forest disturbance activities such as selective logging, firewood extraction, and charcoal production by local communities [19,46]. The results of this experiment support these findings and indicated low agreement between forest disturbance monitoring through remote sensing and ground data. Therefore, data acquired through local communities can help to verify remote sensing estimates and to signal new changes (even before the remote sensing data are available). Essential information such as location, time, area and type of the forest change events provided by local people can be integrated with remote sensing observations to develop near real time forest monitoring systems. Furthermore, even a proof of “no change in forest status” is an important finding to ensure that new activities do not negatively affect the carbon performance in REDD+ implementation. Thus, while remote sensing techniques are the main tools used at the national level to detect deforestation, local level community data can be an important input for analysis of deforestation and degradation events. Moreover, the findings also suggest that local community members can acquire large amount of data at relatively low cost.

### 4.2. Advantage of the Mobile-Device System

Traditionally, forest monitoring data are collected through paper based methods where paper forms are filled in with manually collected data. This leads to difficulties in data translation, digitization and handling, resulting in a lack of confidence in locally collected data. Compared with paper based methods, the proposed system has the capability of automatically capturing a larger variety of data types such as Geo-location, date, text, audio, video and images through smart phones and PDA devices, adding more flexibility in data collection at the community level.

Cyber Tracker has been widely used under ‘Kyoto: Think Global Act Local’ (KTGAL)–research programme in mapping, measuring and monitoring forest carbon services [26]. It has the functionality of a user-friendly form designer and data synchronization over web. Compared to CyberTracker, the system developed in this study provides easy to add features and low cost of deployment facilities. Furthermore, the proposed system reduces difficulties in data translation and digitization, and reduces the time lag for data to be available for national usage. User friendly data transmission features of the system enable the local community to feed the data directly to the database server.

### 4.3. Limitations of the Mobile-Device System

The proposed system also faces some technical challenges. In general, one major drawback of this system is that it works only on Android based mobile phones. The second general issue is maintaining battery power for mobile devices in remote areas; nevertheless this problem can be now easily be resolved by using solar-powered mobile device chargers, which are now widely available at low cost. The third is that this approach allows data storing locally on phones which may get lost or damage. One possible solution for this problem is to use higher storage capabilities inside the phone and transfer data from mobile device to a local computer or storage device on a regular basis. The fourth is data entry errors: it is noticed that local people are more accurate in entering information through a sleeting icon or a check box than through manual entering of text or numbers. The interface design may allow reducing this error. The fifth drawback is the cost. Initially, national support is needed to setup the system but the test case of this study shows that the digital data collection through local community offers cheaper and more timely data than the data collected by the experts, while reaching a comparable accuracy. Finally, it can be observed that there is need of technical supervision, trouble shooting and capacity building program for the local community in order to increase the reliability of the results.

## 5. Conclusions and Future Outlook

In this paper, we have presented an integrated mobile based data collection system designed for local communities to support forestry data collection for their national REDD+ program. The use of mobile device is a very promising field with exponentially increasing number of sensors and opportunities available in the marketplace and has the potential to increase the efficiency of forest monitoring systems. A prototype application for the community based monitoring was successfully developed and implemented in an ethnic minority community of Vietnam. The proposed system was able to facilitate data acquiring, storing, transmitting and displaying by local people.

In order to achieve an added value for CBM, system performance was evaluated in terms of data accuracy and cost. The comparison of community acquired forest inventory data and estimated aboveground biomass with professional expert measurements showed that the local communities are able to acquire data with accuracy comparable to data acquired by an expert, but against lower costs. Furthermore, the disturbance monitoring activity of local people was examined with high resolution SPOT image. The results confirm that communities are more effective than remote sensing to monitor small scale forest degradation due to, for example, subsidence fuel wood collection or selective logging.

The presented system is able to support the acquisition of CBM data that can be directly linked to national MRV in the prospect of data demand, supply, management, reporting and quality assurance. Further development of the presented approach will focus on the establishment of an integrated two way data synchronization system for exchange of information between client and server. In such systems, the acquired data will be linked with cloud computing and it will be possible to deliver near real-time forest monitoring services.

## Figures and Tables

**Table 1.** Comparison of Mobile data transmission means (adopted from [32,33]).

**Mode of data transfer**	**Data types**			**Data volume**	**Cost**

	**Number**	**Text**	**Multimedia**		

SMS	Yes	Yes	No	Low	Low
MMS	Yes	Yes	Yes	Low	Medium
Bluetooth	Yes	Yes	Yes	Medium	Free
3G internet	Yes	Yes	Yes	High	Medium
USB cable	Yes	Yes	Yes	High	Free

**Table 2.** Available SPOT 5 image properties.

**Bands**	**Spectral ranges**	**Ground resolution**
Green	0.50–0.59 μm	2.5 m
Red	0.61–0.68 μm	2.5 m
Near infrared	0.78–0.89 μm	2.5 m

**Table 3.** Evaluation matrix of data entry types and costs.

**User type**	**Educational Level**	**Time for training** **(Hours)**	**Accuracy of data entries (%)**			**Cost of data acquisition** **($ per ha)**
			**Text/Number** **(Manual entry)**	**Capturing Multimedia**	**Text/Number** **(Selection option)**	
Local community	Pre-secondary	4	72	89	100	1.20
Local expert	Secondary-University	4	82	95	100	3.20
National expert	University	4	93	100	100	6.40

**Table 4.** Delay in capturing forest disturbance signal by SPOT image.

	**Forest disturbance signal captured by SPOT**			
Forest disturbance captured by local communities	Date	Detected on same year	Delayed detected (up to 2 year)	Not detected
	2007	16%	48%	36%
	2008	33%	53%	14%
	2009	33%	47%	20%
	2010	65%	20%	15%
